# Biological and mechanical interplay at the Macro- and Microscales Modulates the Cell-Niche Fate

**DOI:** 10.1038/s41598-018-21860-6

**Published:** 2018-03-02

**Authors:** Udi Sarig, Hadar Sarig, Aleksander Gora, Muthu Kumar Krishnamoorthi, Gigi Chi Ting Au-Yeung, Elio de-Berardinis, Su Yin Chaw, Priyadarshini Mhaisalkar, Hanumakumar Bogireddi, Seeram Ramakrishna, Freddy Yin Chiang Boey, Subbu S. Venkatraman, Marcelle Machluf

**Affiliations:** 1 School of Materials and Science Engineering (MSE), Nanyang Technological University (NTU), Singapore, Singapore; 2 Department of Mechanical Engineering, National University of Singapore (NUS), Singapore, Singapore; 3 Faculty of Biotechnology and Food Engineering, Technion—Israel Institute of Technology (IIT), Haifa, Israel

## Abstract

Tissue development, regeneration, or *de-novo* tissue engineering *in-vitro*, are based on reciprocal cell-niche interactions. Early tissue formation mechanisms, however, remain largely unknown given complex *in-vivo* multifactoriality, and limited tools to effectively characterize and correlate specific micro-scaled bio-mechanical interplay. We developed a unique model system, based on decellularized porcine cardiac extracellular matrices (pcECMs)—as representative natural soft-tissue biomaterial—to study a spectrum of common cell–niche interactions. Model monocultures and 1:1 co-cultures on the pcECM of human umbilical vein endothelial cells (HUVECs) and human mesenchymal stem cells (hMSCs) were mechano-biologically characterized using macro- (Instron), and micro- (AFM) mechanical testing, histology, SEM and molecular biology aspects using RT-PCR arrays. The obtained data was analyzed using developed statistics, principal component and gene-set analyses tools. Our results indicated biomechanical cell-type dependency, bi-modal elasticity distributions at the micron cell-ECM interaction level, and corresponding differing gene expression profiles. We further show that hMSCs remodel the ECM, HUVECs enable ECM tissue-specific recognition, and their co-cultures synergistically contribute to tissue integration—mimicking conserved developmental pathways. We also suggest novel quantifiable measures as indicators of tissue assembly and integration. This work may benefit basic and translational research in materials science, developmental biology, tissue engineering, regenerative medicine and cancer biomechanics.

## Introduction

Every tissue can be regarded as a collective of two basic components: cells and their external microenvironment (i.e., niche) including interactions with the extracellular matrix (ECM) and other cells, which occur at the micro and nano scales^1^. Cell–ECM communications are governed by reciprocal biomechanical, structural, and biochemical interactions^1,2^. Particularly, many studies show that ECM biomechanical properties greatly affect cell behavior and function, for instance via mechanotransduction^3–5^—translation of external mechanical forces into electrochemical activity—promoting changes in cell shape, size and differentiation states (recently reviewed in)^6,7^. Much less, though, is known about how cells affect their external niche biomechanics. The biomechanical cell contribution to tissue generation through development, when engineering substitute tissues *in vitro* or during regeneration following injury, is a complex process comprising various cell types, ECM compositions and interaction levels^8^. It is, therefore, challenging to identify, map and quantify the relative contribution of each factor involved in the biological and mechanical dynamics of tissue formation. Furthermore, conventionally employed gross mechanical characterization methods produce average tissue values, which may fail to reveal the subtle temporal and spatial distribution of restricted cell influences (i.e., at the microscale) on their local niche biomechanics.

To reduce the complexity associated with such multifactorial parameters and studies, there is a crucial need to develop appropriate *ex vivo* models that can mimic physiological-like cell–ECM interactions under defined conditions. The ideal model should take into account the choice of the ECM mimicking biomaterials as well as that of representative cell types used. The biomaterial utilized for modeling purposes should be bioactive, enabling physiological-like cell communications, while matching the mechanical properties of the mimicked tissue. For instance, most biological tissues are considered viscoelastic, that is combining elastic (linear stress-strain relationship) with viscous (exponentially decaying stresses through time) behaviors^9^. Further, both cells and ECM exhibit a unique characteristic of strain stiffening—during which the elastic modulus (slope of the stress-strain curve) increases non-linearly with the increase in applied strain^2,10,11^. These complex biomechanical properties, naturally occurring in biological tissues, however, are not easily mimicked using synthetic production methods, and usually requires the application of biologically derived materials^10^. Last, the choice of cells to study such interactions should be relevant to the tissue of interest at the studied developmental stage. For example, two predominant cell–ECM interaction types that exist in many tissues and can be utilized for basic model characterization studies are: polarized endothelial/epithelial cells commonly coating basement membranes, and interstitial integration of supportive mesenchymal cells within 3D fibrous ECM structures^2^.

One possible way to model cell–ECM interaction involves the use of decellularized ECM materials^1^. Decellularization protocols have been specifically designed to remove all cells from a source tissue resulting in isolation of biologically active, cell supportive composite biomaterial that comprises the unique ECM makeup of the source tissue^12^. Such decellularized ECM often displays comparable mechanical characteristics to its source tissue, and is amenable to cell remodeling and biological crosstalk much like in its native environment during homeostasis and regeneration^8^. Consequently, when decellularized ECM is repopulated with cells *in vitro*, it can provide a robust platform for the studies of biomechanical cell-ECM interplay during tissue development.

We, therefore, studied such cell contribution to our developed decellularized porcine cardiac ECM (pcECM)—a representative soft tissue ECM— exhibiting cell support ability^13–17^, bioactivity^18,19^ and biomechanical matching properties to its native tissue of origin^9,14,20^. In this study, we hypothesized that pcECM can serve as an *ex vivo* model to study the different biomechanical contributions of reseeded cells during early tissue formation and integration. Understanding such contributions may advance future therapeutic outcome of the engineered tissue. For these studies, the gross (tensile tester) and localized (atomic force microscopy, AFM) distribution of the acellular and reseeded constructs’ mechanical properties as well as the native left ventricular tissue (as control) were evaluated. For reseeded constructs human umbilical vein endothelial cells (HUVECs), human mesenchymal stem cells (hMSCs) and co-cultures thereof were used, representing a spectrum of predominant cell–cell and cell–ECM interactions occurring in soft tissues. Finally, histology, cell expression phenotyping and scanning electron microscopy (SEM) were performed, suggesting significant and important, yet different roles for each cell type in tissue formation and the synergistic effect of both towards cell–tissue integration and maturation. Accordingly, this study establishes a new model system to study *in vitro* cell–niche interactions with clear implications for basic stem cell, developmental biology, and materials science research, as well as translational research in tissue engineering and regenerative medicine.

## Results

### pcECM production and microscopic characterization of cellularization

**Figure 1:**
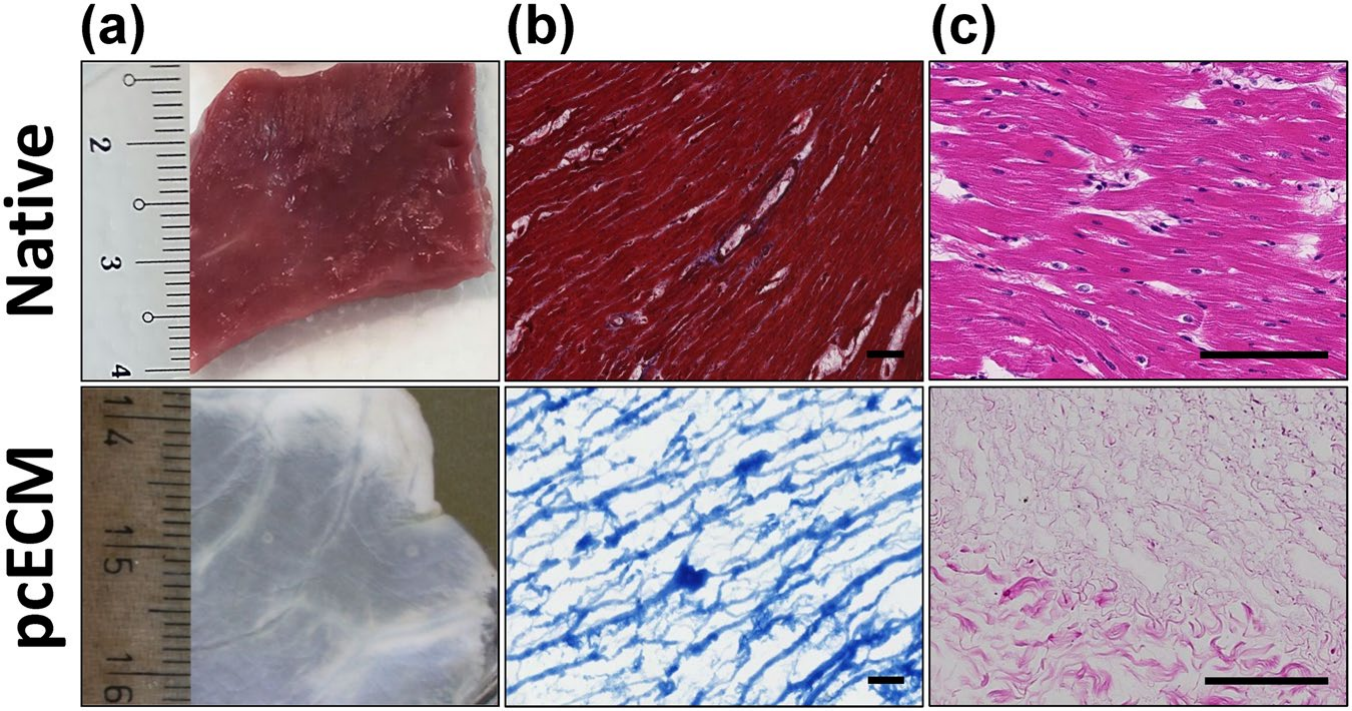
Macroscopic and microscopic views of native and decellularized tissue constructs. Native left-ventricular slab of porcine origin and left ventricular derived decellularized pcECM scaffold (a, upper and lower panels, respectively). The pcECM is heterogeneous and comprises areas of dense collagen (stronger white color) adjacent to more spacious ECM (pale shades). Masson’s trichrome (MTC) staining of native and pcECM matrices demonstrating the effectiveness of the decellularization (lack of red color attributed to cells) and the degree of ECM fiber ultrastructure preservation (in blue, b, upper and lower panels, respectively). Hematoxylin and eosin (H&E) staining of native and pcECM matrices (c, upper and lower panels, respectively). Representative images are presented out of n = 5 samples in each group and at least three different regions of interest (ROI) images in each case. Scale bar: 100 µm.

**Figure 2:**
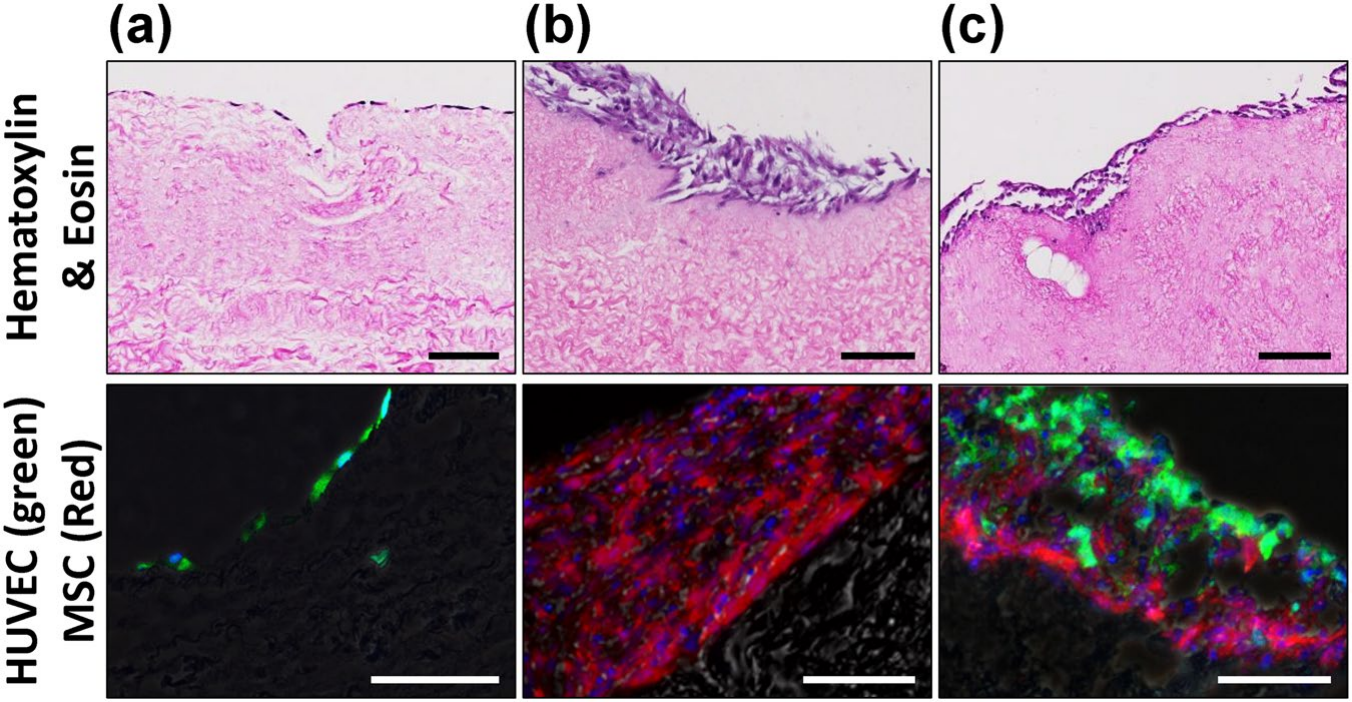
Distinct tissue formation morphologies are cell–culture and cell-type dependent. Representative H&E and fluorescent microscopy images of tissue cross-sections after three days of cultivation on the pcECM are shown as indicated for HUVECs culture alone (**a**), hMSCs culture alone (**b**) and their co-culture (**c**). The fluorescent images show HUVECs stably expressing green fluorescent protein (GFP) in green; and hMSCs stably expressing red fluorescent protein (RFP) in red. Scale bars, 100 µm.

**Figure 3:**
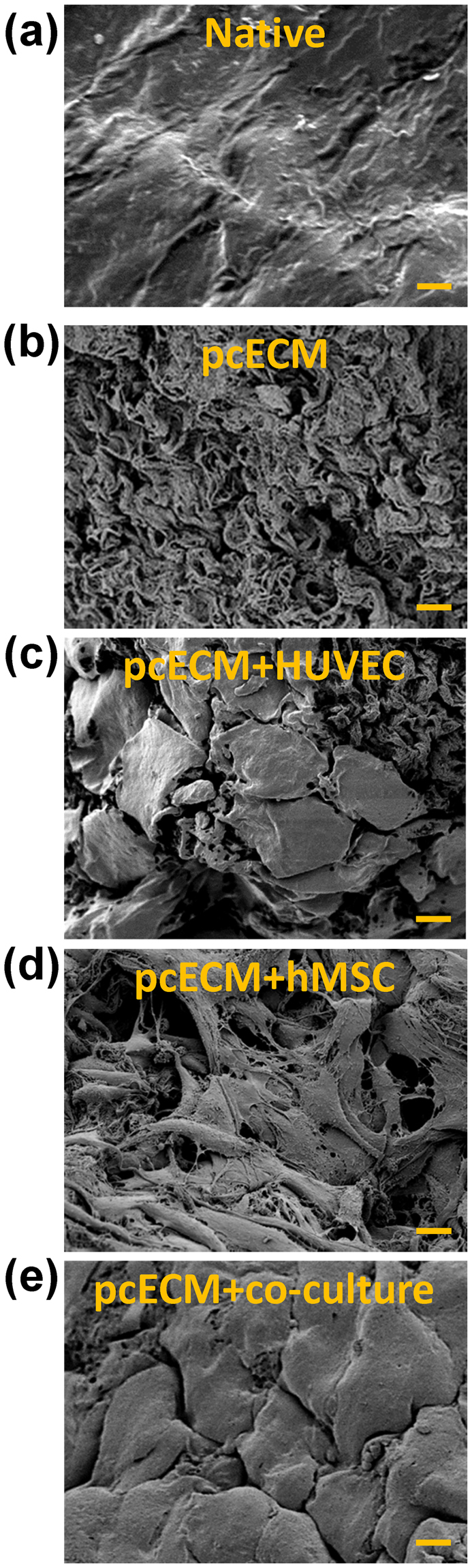
SEM images of pcECM, recellularized and native tissue constructs. Sample attribution is as indicated in (**a**–**e**). Representative images are presented out of n = 5 samples per group and at least three regions of interest taken for each case. While pcECM has a more porous morphology (**b**) than the native tissue (**a**), HUVECs recellularization resulted in a typical ‘cobble-stone’ like morphology coating the surface of the pcECM (though not homogenously, some areas still show bare and porous ECM-like features, (**c**). hMSCs on the other hand, show a more ECM-interactive spindle shape morphology (**d**) The co-cultured surface (**e**) resembles the HUVECs cobble stone morphology (**c**) albeit with an apparent higher concavity which may indicate a different underlying interaction. Scale bars: 10 µm.

Successful decellularization of native porcine ventricular tissue (Fig. 1, upper panel) yielded completely acellular pcECM (Fig. 1, lower panel), as verified both macroscopically by material blanching (Fig. 1a), and microscopically by Masson’s trichrome (MTC) and Hematoxylin and eosin (H&E) stains (Fig. 1b,c, respectively). Effective recellularizing of the decellularized pcECM matrices with both mono- and co-cultures of HUVECs and hMSCs, serving as model cells was achieved. Histological cross-section analyses revealed that, three days after seeding, HUVECs formed a cell-monolayer, coating the pcECM surface (~2–4 µm in depth Fig. 2a)—as opposed to penetration and integration of hMSCs up to ~100 µm deep into the pcECM (Fig. 2b). Based on these marked morphological differences between the mono cultures of each cell type alone, we speculated that a simultaneous co-culture of both cells (pre-mixed at a 1:1 ratio) would result in an intermediate phenotype. We, therefore, expected such co-culture conditions to result in mixed HUVECs and hMSCs presence within the same ~100 µm depth, a distance that was previously reported as a diffusion limitation for static culture conditions using the same pcECM material^15^. Surprisingly, the co-cultured cells appeared, instead, to be interacting and self-organized in ‘primitive-streak’-like layering morphology, such that the HUVECs, remained mostly on the surface, while hMSCs remained ‘sandwiched’ at the interface between the HUVECs and the pcECM bulk (Fig. 2c). Surface analyses using scanning electron microscopy (SEM) further confirmed these observation (Fig. 3). While acellular pcECM comprised compact porous sponge-like structure (Fig. 3b), HUVECs (Fig. 3c) and hMSCs (Fig. 3d) recellularized pcECMs displayed both bare pcECM-resembling areas, as well as morphologies of dense coating layers attributed to stretched cells (similar to the native tissue, Fig. 3a). The cell layer morphologies of HUVECs and hMSCs, however, were different, as HUVECs arranged in a typical cobblestone-like monolayer while the hMSCs displayed elongated fibroblastic-like spindle shaped structures that interconnected with the pcECM. Interestingly, in the co-culture system, the predominant surface morphology was similar to that of HUVECs, with a slightly more concave appearance (Fig. 3e), further corroborating our histological findings and suggesting that HUVECs too, responded to their interaction with the underlying hMSCs. The observed cell self-organization could potentially enable the ‘sandwiched’ hMSCs’ to receive both pcECM and HUVECs cell–cell communication signaling from opposite ends but in parallel, leading to differential gene expression regulation as compared to the mono cultures alone. We were particularly interested in the effects such self-assembly and differential gene expression might have on early tissue formation, remodeling and integration processes. We, therefore, further conducted a systematic analyses of both the mechanical properties of the reseeded constructs (macro- and microscopically) as well as qPCR arrays at the mRNA level to evaluate such possible effects.

### Macroscopic biomechanical characterization suggests partial mechanical properties restoration by recellularization of the pcECM

**Figure 4:**
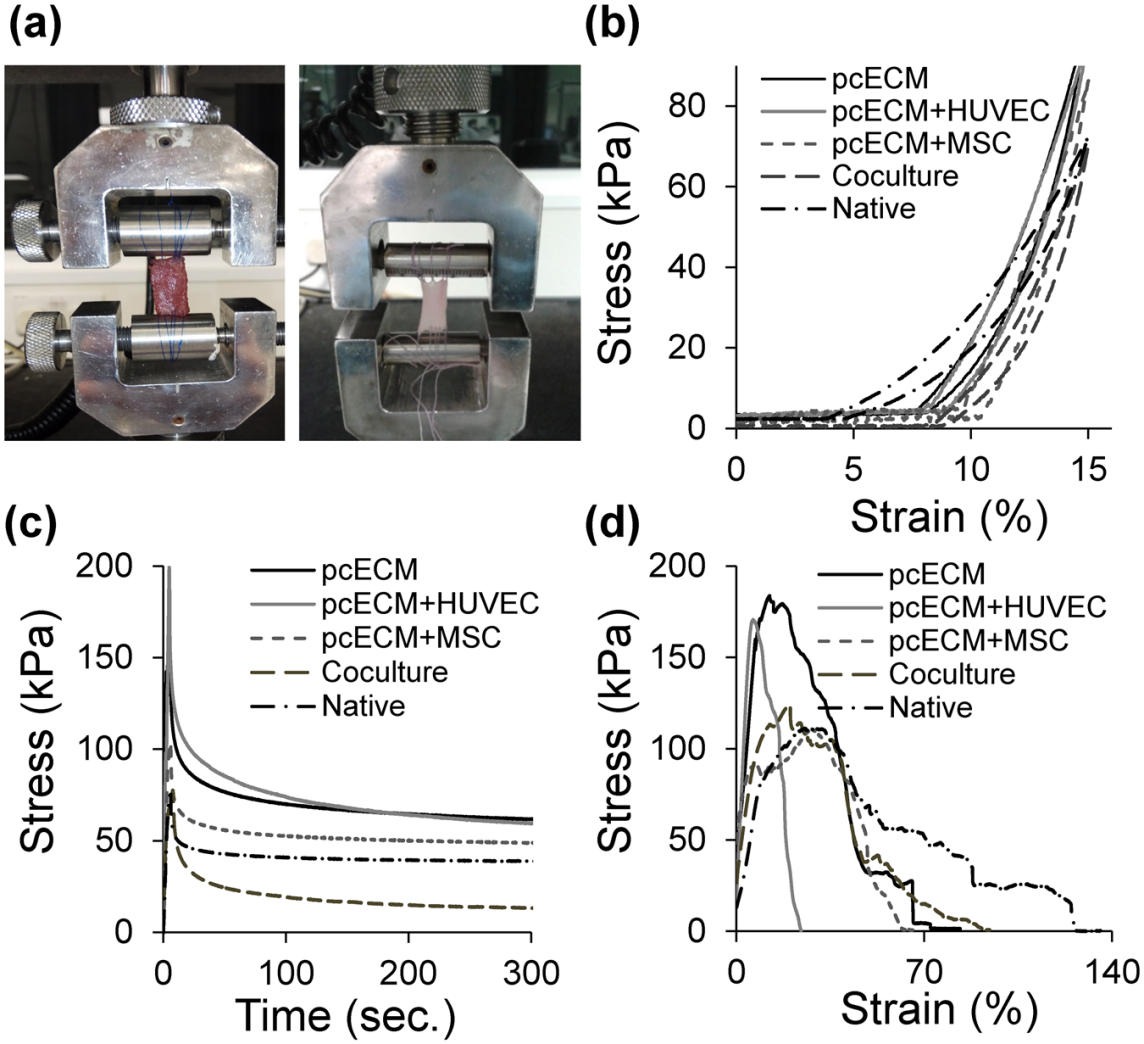
Macroscopic uniaxial mechanical evaluation of the pcECM (acellular and recellularized) exhibit viscoelastic behavior with strain stiffening similar to native ventricular tissue. Sample mounting is demonstrated for native tissue (**a**, left image) and its decellularized counterpart (**a**, right image). Representative curves of the cyclic loading, stress relaxation and tensile failure assays performed (**b**–**d**, respectively). Note that all groups tested displayed viscoelastic characteristics: Strain stiffening (‘’ shaped loading curves), and hysteresis loops—formed between loading and unloading curves at the same cycle (**b**); as well as creep—exponential stress decay over time at constant strain (**c**). These viscoelastic characteristics (strain stiffening and creep), not easily generated using synthetic materials, establish the biomechanical suitability of the pcECM for modeling early soft tissue formation processes also at the biomechanical level. Quantifying macro-scale average parameters derived out of these assays and their statistical comparison for the various groups tested appear in Table 1.Statistical significance is observed in some of these parameters for the native and acellular pcECM groups. While recellularization generally appears to restore the biomechanical profiles of pcECM towards the native state, the intergroup significance between various culture conditions is not achieved nor is it particularly informative at this time point using these macroscopic testing assays. Attempts to perform these tests at later time points were non successful due to cellular remodeling and shrinking of the reseeded sample (data not shown).

Our *macroscopic* mechanical properties testing employed an Instron machine (Fig. 4a) and comprised three sequential assays: cyclic-stress (Fig. 4b), stress-relaxation (Fig. 4c) and strain-to-break (Fig. 4d). These assays demonstrated viscoelastic properties (strain-stiffening and creep behaviors, as detailed below) for all tested groups serving a twofold purpose: basing the suitability of the pcECM as a model material; and potentially identifying significant key gross biomechanical differences between culture conditions (Table 1).

**Table 1:** Summary of macroscopic (Instron) mechanical testing results*.

Assay	Parameters	pcECM	Native	HUVECs	hMSCs	Co-culture
Stress relaxation	Maximal tensile stress (kPa)	164 ± 15^a,b^	103 ± 20^c^	219 ± 25^a^	96 ± 33^b,c^	72 ± 10^b,c^
	ΔTensile stress during relaxation (kPa)	116 ± 22^a,b^	44 ± 10^c^	145 ± 5^a^	67 ± 37^b,c^	62 ± 16^b,c^
	Time at Δrelaxation of 0.5 N (sec)	2 ± 2^a^	3 ± 3^a^	0.5 ± 0.3^a^	4 ± 3^a^	1.0 ± 0.5^a^
Tensile failure	Young’s modulus (MPa)	1.9 ± 0.1^a^	0.7 ± 0.3^b^	2.4 ± 0.6^a^	1.5 ± 0.7^a,b^	1.2 ± 0.1^a,b^
	Yield stress (kPa, at 0.2% offset)	136 ± 36^a^	81 ± 50^a^	114 ± 30^a^	75 ± 22^a^	70 ± 30^a^
	Ultimate Tensile Stress (kPa)	198 ± 28^a^	117 ± 28^b^	136 ± 55^a,b^	119 ± 30^b^	106 ± 34^b^
Cyclic loading	Maximal stress at 15% strain (kPa)	76 ± 21^a^	70 ± 5^a^	87 ± 17^a^	57 ± 30^a^	69 ± 5^a^
	Hysteresis energy dissipation (kJ/m3)	0.40 ± 0.13^a^	0.58 ± 0.16^a^	0.63 ± 0.18^a^	0.55 ± 0.38^a^	0.47 ± 0.37^a^

(*) Identical uppercase letters represent statistically significant groups in each row whereas values that do not share the same uppercase letter are significantly (*p* < 0.05) different as determined by one-way ANOVA with Tukey’s honest significant difference (HSD) post hoc correction for inflated type I error.

The cyclic-stress assay (Fig. 4b) assesses the tissue construct ability to withstand fatigue during repeated ‘working’ cycles of loading (in the direction of increasing strains) and unloading (opposite direction of decreasing strains). For viscoelastic materials, cyclic stress assays typically result in a right shift of the unloading- relative to the original loading curve, due to energy loss during the process. The area blocked within the so called ‘hysteresis loop’ (a loop shaped area between the loading-unloading curves of each cycle) is equivalent to the energy dissipation of the material during the stress cycle. Three days post seeding, all samples displayed similar strain-stiffening manifested by upwards-bended loading curve () with viscoelastic hysteresis (Fig. 4b), having no significant differences between groups in the average energy dissipation and maximal stress reached at 15% strain (Table 1).

Another hallmark of viscoelastic materials is that of creep—the tendency of a solid material to move or deform slowly under the influence of mechanical stresses. Using the stress-relaxation assays, samples were stretched and held at a constant strain for at least 300 seconds during which the stress exerted by the material on the Instron machine was measured and plotted as a function of time. Creep is manifested by an exponential decay in the stress values through time in the constant strain region. Our results indicate that all tested samples exhibit force-decay over time within similar time frames (stress relaxation assays, Fig. 4c and Table 1). The maximal tensile stress reached and the change (delta) in stress during relaxation, however, were significantly higher for the acellular pcECM than that measured for the native tissues (Table 1). Stress values and their relaxation delta in HUVECs-reseeded constructs remained similar to the unseeded pcECM and were significantly higher than in native tissue. Interestingly, these values for hMSCs and co-culture reseeded constructs were intermediate between the acellular pcECM and the native tissue, being statistically similar to both. Thus, the maximal tensile stress and the stress delta during relaxation, could potentially serve as macroscopically gross markers of the biomechanical outcome of recellularization using the different culture conditions employed. However, the sensitivity of the assay in our hand was not sufficient at this time point to allow for statistically significant recognition of cell-type dependent biomechanical contributions.

A similar trend was apparent also using the ‘strain-to-break’ assay. The Young’s moduli (slope of the linear portion of the stress strain curve) and the ultimate (maximal) tensile stress (UTS) reached prior to samples’ failure displayed significantly higher values for acellular pcECM than for native tissues (Fig. 4d, Table 1). Restoration of the Young’s moduli and UTS values towards those of the native state were cell-type dependent, though, and necessitated the presence of hMSCs in the culture (either alone or together with the HUVECs).

### Microscopic biomechanical characterization reveals bi-model elasticity distribution profiles and a synergistic effect for co-cultures in tissue formation, integration and maturation

**Figure 5:**
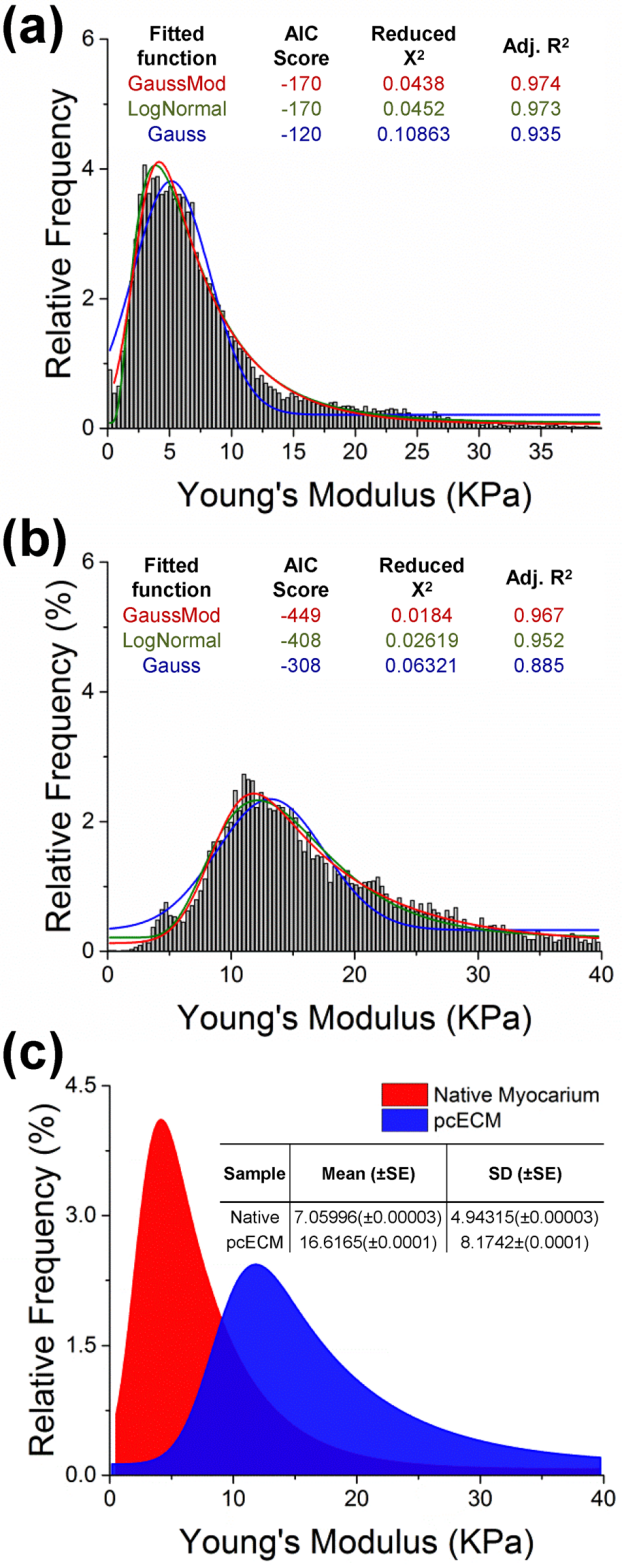
Young’s modulus distribution model-fitting. Native tissue (**a**) and decellularized pcECM (**b**) representing raw data fitting results. In each case, the best three fitting models are shown ranked using the Akaike information criterion (AIC), χ^2^ goodness of fit and adjusted R^2^ values (OriginPro 2015 16 bit Sr2 b9.2.272, OriginLab Corporation, Northampton, MA). Successful fit was considered as having the minimal AIC value with a χ^2^ < 0.05 and adjusted R^2^ > 0.95. Among all models tested, the exponentially modified Gauss model (GaussMod) was found to provide the optimal fit for all samples and was, therefore, used for subsequent data analyses and interpretation. GaussMod Fitted functions (representative native and pcECM are shown one next to the other as indicated in (**c**) were subsequently used for derivation of descriptive statistics and all analyses and comparisons made.

**Figure 6:**
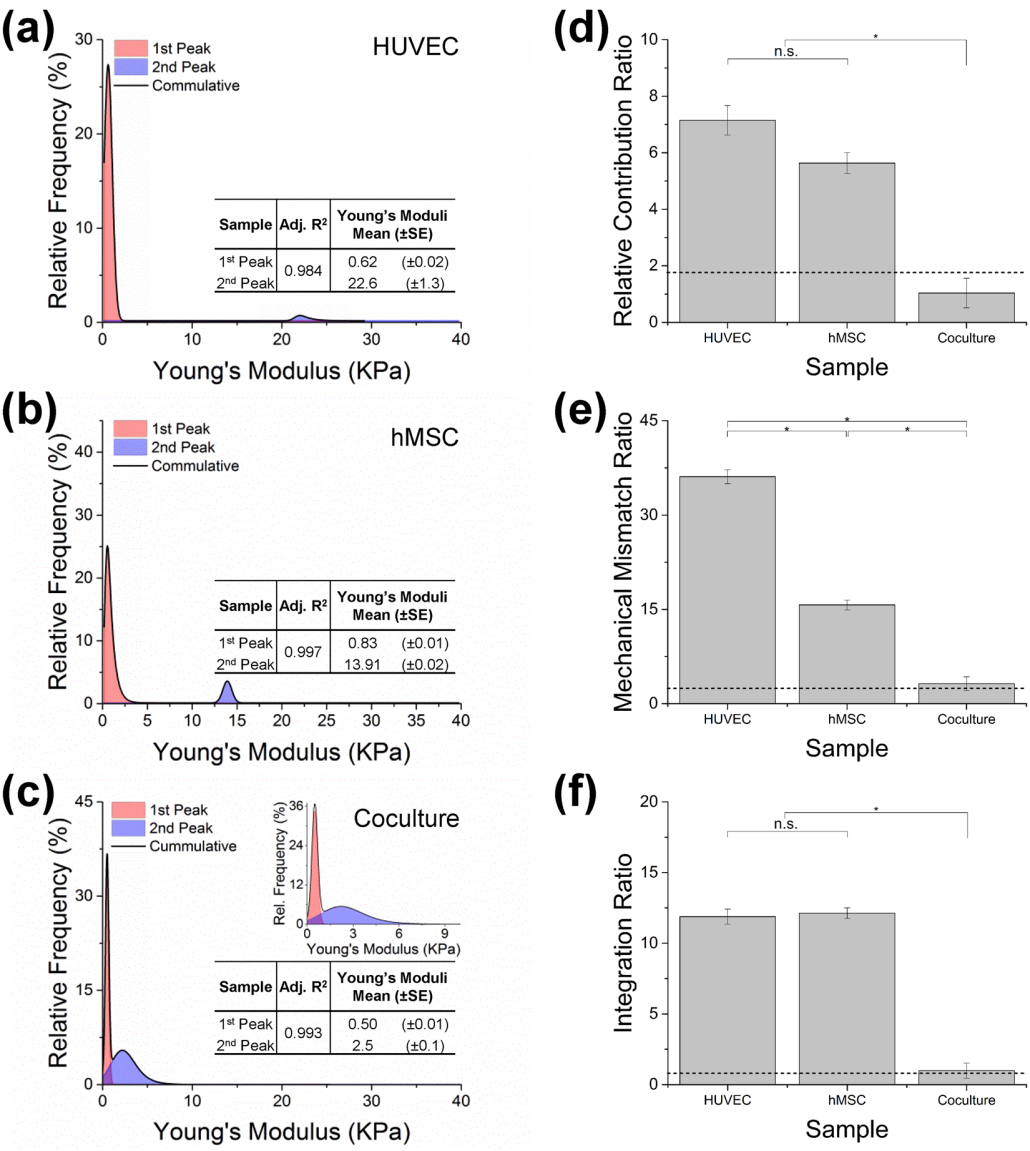
Bi-modal GaussMod Young’s modulus distributions and inter-peak descriptive statistical relations as indicators of tissue formation and cell–ECM biomechanical integration. GaussMod fitting bi-modal curves of Young’s modulus distribution measured for HUVECs alone (**a**), MSC alone (**b**) and their co-culture (**c**), three days post pcECM seeding. The red peak is attributed to known cell Young moduli values, whereas the blue peak corresponds to pcECM contribution. The ECM:cell relative contribution ratio (RCR, **d**), their mechanical mismatch ratio (MMR, **e**), and the integration ratio (IR, **f**) between cell and pcECM attributed peaks within the overall biomechanical profile displayed was quantified for n = 5 biological samples per group. Dashed lines in d-f represent the corresponding ECM:Native tissue ratios in each case for comparison reasons. (*) represents statistical significance (*p* < 0.05) as assessed using one-way ANOVA with Tukey’s post hoc correction. (n.s.) denotes non-significant difference.

AFM was used to better understand the mechanical properties of the newly formed tissue at the microscale. The AFM system set-up and data acquisition are described in the Supplementary Data and Supplementary Fig. S1. The distributions of locally mapped Young’s moduli from randomly selected representative areas across all samples are shown in the histogram modeling graphs of Figs 5 and 6.

To analyze the underlying biomechanical contributions of cells and pcECM to tissue-like organization, statistical modeling was initially employed for the control groups—native ventricular tissue and acellular pcECM data. Characterization of the native tissue Young’s modulus histograms revealed a single peak, right-skewed distribution, which was well fitted by both log-normal and exponentially modified Gauss (GuassMod) models, but displayed poorer fit for the standard normal model (Gauss, Fig. 5a). Similar modeling of the Young’s modulus distribution in acellular pcECM (and also reseeded pcECM samples) revealed the best fit for the exponentially modified (GaussMod) model fitting (Figs 5b and 6), which was subsequently used to produce descriptive statistics for all experimental groups tested.

Comparing the distributions of both acellular pcECM and native tissue, it is evident that the pcECM is stiffer i.e., having higher Young’s moduli values, also at the micro level (Fig. 5c), as it lacks the cellular contribution to viscoelasticity that can be seen in native tissue. Three days after seeding, the reseeded pcECMs Young’s moduli for HUVECs (Fig. 6a), hMSCs (Fig. 6b) and their co-culture (Fig. 6c) revealed in all cases bi-modal distributions. The first peak values (P_1_ in red) were substantially lower and narrower than those measured for both bare pcECM and native tissue (Fig. 6), and corresponded to known values of cell elasticities cited in the literature^21,22^. The second peak (P_2_ in blue) is attributed to stiffness contributed by the pcECM component of the reseeded construct; however, the second peak area (‘A’) and relative location were cell-type dependent. From the statistical fit data, we suggest the calculation of three descriptive and characteristic parameters that can be useful in quantification when describing the different relationships between the two peaks for each sample type (Fig. 6d–f). The three descriptive statistics developed are the ‘Relative Contribution Ratio’ (RCR), ‘Mechanical Mismatch Ratio’ (MMR) and ‘Integration Ratio’ (IR) between the major cell contribution peak (P_1_) and the relative ECM contribution peak (P_2_). The mathematical definitions of these variables are detailed in the Supplementary Data.

As AFM measures the surface biomechanical properties, HUVECs coating of the pcECM surface area (Fig. 2a) is represented by the relative peak area-under-curve ratio—that is the RCR of both components (cells–P_1_ and pcECM–P_2_, Fig. 6a). We referred to the relative native tissue to pcECM area-under-curve-ratio (A_native_/A_pcECM_, Fig. 6) as a good approximation of the RCR of cells-to-surface interaction/coating in mature natural tissues (dashed line as reference in Fig. 6d). In contrast to HUVECs repopulated constructs, hMSCs presence appeared to reduce the average (i.e., location of peak center) and SD of the entire second peak (P_2_) distribution towards the first peak (P_1_, Fig. 6a,b and e). This phenomenon indicates a reduction in the overall MMR between the cells and the pcECM towards (though still significantly higher than) the MMR values measured in the native tissue (Fig. 6e). The apparent pcECM remodeling induced by hMSCs was concomitant to cell penetration of at least 100 µm deep into the pcECM scaffold (Fig. 2b). Despite this effect by hMSCs, both mono-cultures (HUVECs and hMSCs alone) appeared to be less integrated with the tissue construct at that same time point—as the peaks of each components remained separately segregated (IR, Fig. 6f). Interestingly, a co-culture of both cell types displayed synergistic and faster tissue maturation—graphically manifested by overlapping peaks—as indicated by all three parameters that were reaching levels similar to those of native tissue (Fig. 6d–f).

### pcECM induces a different remodeling expression profile in a cell-type dependent manner

**Figure 7:**
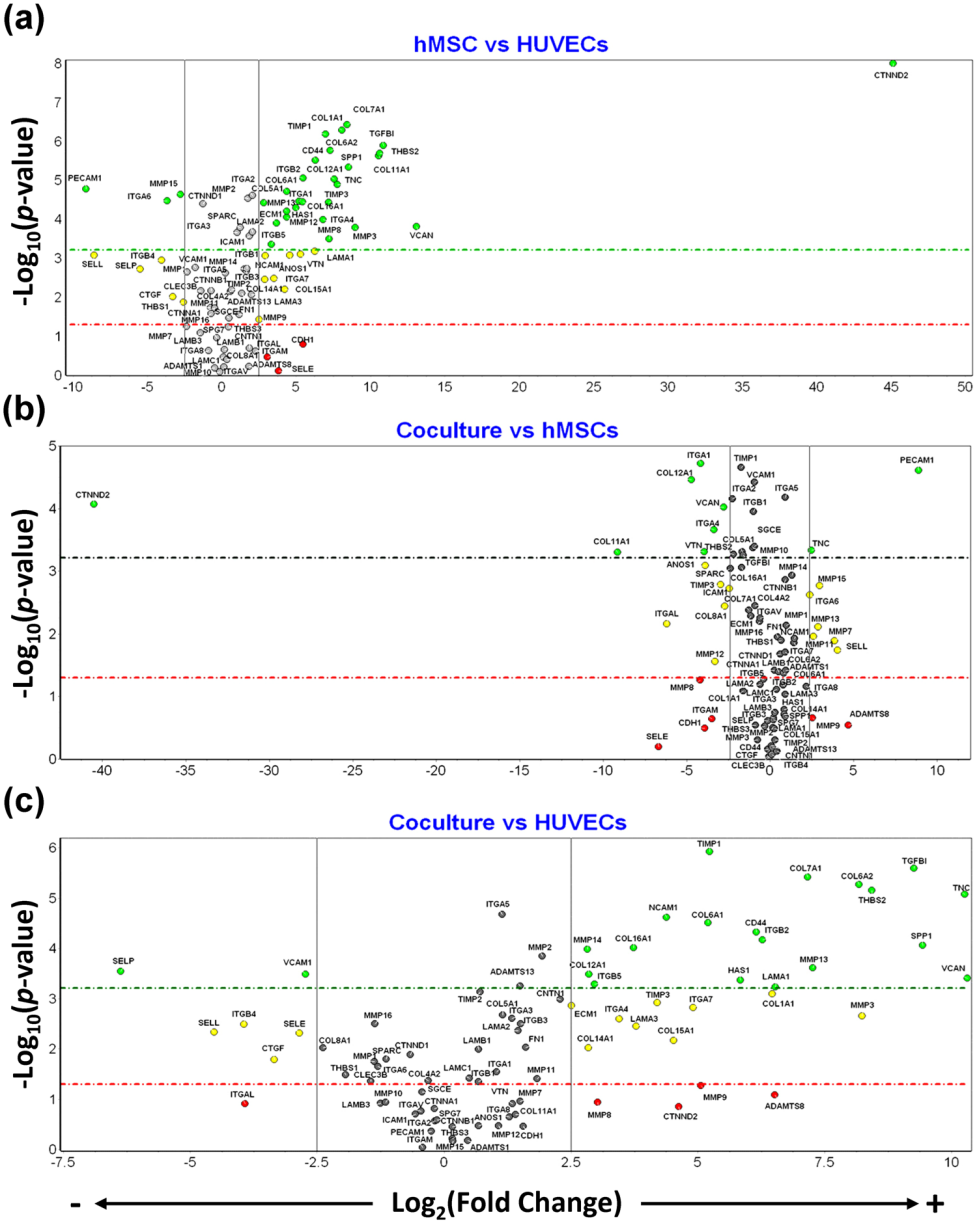
Distinct cell–ECM interactions during tissue formation are cell–culture and cell-type dependent. Volcano plots showing the significant *p*-value levels for each ECM remodeling related gene as a function of its expression fold-regulation on pcECM. Each gene fold-regulation was normalized first to its own sample housekeeping gene expression level, then to equivalent normalized expression of identical three-day cultures on plates to identify which expression is pcECM induced. These double normalized expressions were then used to compared between the three culture systems to generate the three volcano plots as indicated (hMSC vs. HUVECs monoculture, (**a**); co-culture vs. hMSCs, (**b)**; and co-culture vs. HUVECs, (**c**). Positive fold-regulation values show upregulation in the first culture system of each comparison pair while negative values show downregulation (or upregulation in the second culture system compared). Green marked genes showed both high statistical significance (*p* < 0.00061 per individual T-test based on ANOVA with Tukey–Kramer’s post hoc correction) as well as |X| > 5 fold-regulation change. Red dots are statistically not significant (*p* > 0.05) but have considerable |X| > 5 fold-regulation change; Yellow dots have a considerable |X| > 5 fold-regulation change with *p* values smaller than 0.05 but larger than the Tukey-Kramer’s post hoc correction cut-off value. Grey dots represent genes that are not sufficiently up or down regulated, even if statistically significant. Detailed values and statistical comparison results for all genes appear in Supplementary Tables S1–S3. Only the green dotted groups (six gene clusters of significantly top regulated genes) were used for gene-set analyses and comparison between the different culture conditions.

**Figure 8:**
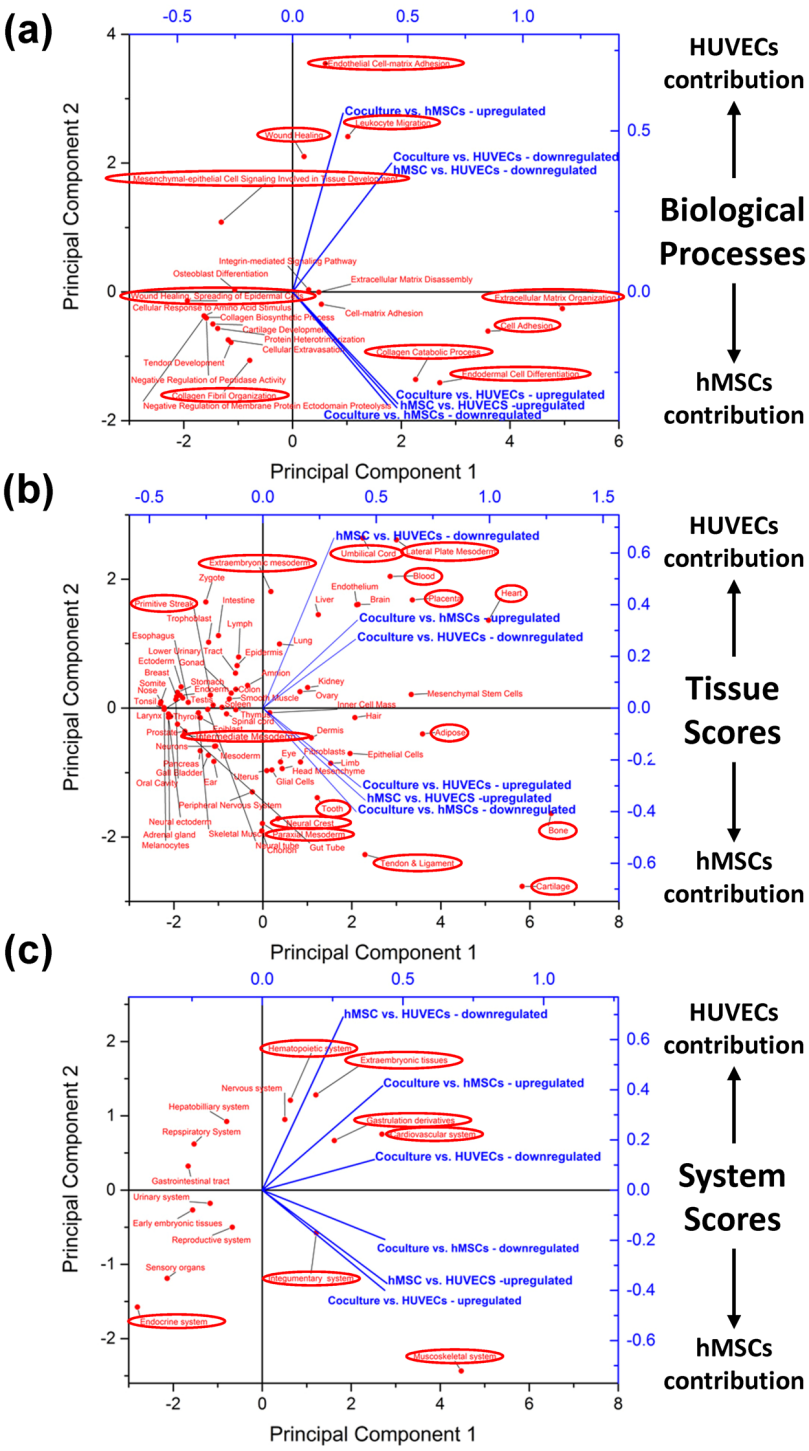
PCA representation of the score vectors and loadings for all significantly top regulated gene clusters based on GeneAnalytics gene set analyses. The six gene clusters identified in Fig. 7 and Supplementary Tables S1–S3 are represented by the blue vectors—each comprising specific culture condition comparison and significantly high fold (>5) regulated gene set. These vectors are ploted using principle component analyses (PCA) based on their expression signature similarity to known biological processes (**a**), tissues (**b**) and organ systems (**c**). The common loadings used for the scoring of these vectors are indicated by the red dots. Generally, the more extreme you get on the ordinate (principle component 2 axis), the closer you get to pure monoculture conditions, as indicated, whereas more central location along the ordinate suggest co-culture involvement. As the vectors represent a superposition of many different pathways, some in contradicting directions, we have circled in red major loadings which position and direction suggest key involvement in positioning of the gene cluster vectors.

To further characterize the differences between the various cell cultures in terms of ECM remodeling and integration, we performed qPCR array analyses for the differential expression of human ECM and adhesion molecules. Forty-eight genes of interest (out of 89 genes tested) were identified as being the top differentially expressed among all samples (*p* < 0.01, ANOVA). Of these genes, different subgroups were significantly up- or downregulated when comparing between the different pcECM seeded cultures (*p* < 0.00061 per individual T-test based on Tukey–Kramer’s post hoc correction, Fig. 7). Genes that were up- or downregulated by at least five-fold (Fig. 7, green marks) were further analyzed using GeneAnalytics to score and classify matching biological processes, tissue and organ systems (Fig. 8). The results indicated that in all cases, the biological process involved relate to ECM organization (Fig. 8a), though, with different implicated organ systems and tissues identified as relevant for each sample type.

Comparing double normalized (to both housekeeping genes and respective plate controls) hMSCs to HUVECs mono-cultures on the pcECM, revealed differing genetic upregulation in each case. Twenty seven genes were upregulated in pcECM-hMSC culture compared to three major genes that were upregulated in pcECM-HUVECs culture (Fig. 7a). Co-culture significantly downregulated six genes (of the 27 upregulated in hMSCs monocultures), and two (ITGA6 and MMP15) of the previously three upregulated genes in HUVECs monocultures, while modifying other gene expression as well (e.g. TNC and VTN, Fig. 7b). Concomitantly, 15 of the 27 genes that were highly upregulated in hMSC monocultures, were also upregulated in the co-culture system, joined by additional three genes (MMP14, VCAM1 and LAMA1, Fig. 7c). Of note is that MMP13—that was mildly upregulated in hMSC monocultures by 20.75 fold (Supplementary Table T1)—jumped to 153.76 positive fold regulation (Supplementary Table S3) suggesting higher ECM remodeling in the co-culture system. Last, the snapshot taken using the qPCR array shows also significant downregulation in the co-culture system of two genes highly expressed by HUVECs monocultures on the pcECM (VCAM1 and SELP, Fig. 7c). The co-culture conditions, thus, indicated an altered gene expression profile of the forming tissue construct, in which HUVECs and hMSCs interactions with the pcECM resulted in a synergistic effect rather than a simple additive one.

To further elucidate the biological processes, tissue and overall organ system types and developmental stages matching the signatures of the differentially regulated genetic networks identified, we performed gene set analyses using GeneAnalytics. This gene set analysis was performed individually for each gene cluster comprising specific culture conditions’ comparison and either positive or negative significant fold regulation (as identified in Fig. 7). The combined scoring results for all gene clusters are statistically presented using principal component analyses (PCA) and explain ~85% of the inherent variabilities using just two principal components (Fig. 8). All gene cluster scores (blue vectors, Fig. 8) seem to be narrowly distributed along principal component 1 (PC1), probably owing to similar balancing influences from opposite ends of this axis (loadings represented by red dots in Fig. 8). For instance, looking at the major biological processes identified, a significant ECM organization processes were occurring towards the extreme positive end of the PC1 axis, probably balanced by wound healing processes on the negative side (Fig. 8a). This result serves as an internal control for this analyses, as it was expected from a panel of ECM remodeling and adhesion molecules qPCR array.

Organization along the second principal component axis (PC2), nevertheless, suggested major HUVECs contribution in the positive direction, while major hMSC contribution was achieved in the reverse (negative) direction. From the spread of the blue vector scores and the red loadings, it seems that generally monoculture only contributions appear towards the extremities of the PC2 axis, whereas co-culture contributions were concentrated more towards the midline. Circles in Fig. 8 point to loadings that seems to highly influence the direction and location of the blue vector lines. Thus from a biological process perspective (Fig. 8a), hMSCs presence in the co-culture significantly contributed to both collagen catabolic processes and collagen fibril organization—representing major tissue remodeling processes. HUVECs in co-cultures, on the other hand, appeared to both downregulate several of the remodeling processes of hMSCs monocultures, while initiating wound healing like processes as well as mesenchymal-epithelial cell signaling involved in early tissue development.

Matching tissue scores have similarly identified hMSCs presence as a genetic expression matching several different mesenchymal lineage tissues (e.g., bone, tooth, cartilage, and tendon & ligament, Fig. 8b). HUVECs presence in the co-culture system enabled both downregulation of mesenchymal linages while adding specific recognition of both the umbilical cord, placenta and heart (from which HUVECs and pcECM were sourced, respectively). Of note is the recognition of several mesodermal lineages (intermediate, lateral plate mesoderm and extraembryonic mesoderm)—all being located on opposite directions from the hMSC specific contributions, as well as the primitive streak located in the center of the PC2 axis. The spreading and relative locations of these mesodermal lineages suggest dependency on interaction occurring between hMSCs and HUVECs rather than simple hMSC contribution to this gene expression. Likewise, while hMSC individual contribution is towards the muscoskeletal system genetic profile, and HUVECs contribute predominantly towards Hemaopoitetic and extraembryonic tissues, the co-culture of both resulted in expression matching gastrulation derivatives as well as specific cardiovascular system recognition (Fig. 8c). Therefore, both tissue and organ system results echo the primitive streak like arrangement observed in our histological analyses of Fig. 2c.

## Discussion

In this study, we have developed a unique model system and methodology to study *ex vivo* cell–niche interactions, at early stages of tissue formation, while emphasizing the mechanical and biological aspects of these relations. Using this model system, we demonstrated that different cell types—interacting with identical bioactive pcECM—contribute uniquely to tissue assembly and maturation, hence affecting the newly formed tissue biological and mechanical properties, and directing it towards distinct phenotypic identities. Our mRNA analyses identified unique expression profiles of co-cultures on the pcECM, resembling both early tissue generation signatures (gastrulation derivatives, intermediate and lateral plate mesoderm associated with epithelial–mesodermal cell signaling during development), and cardiovascular specific recognition matching the pcECM source. This unique expression profile may result from the layered ‘primitive-streak’-like niche self-assembly and organization of co-cultured cells on the bioactive pcECM, identified both histologically as well as through gene set profiling. Several groups have previously reported on the empirical benefits of co-culture in enhancing tissue integration, (for example in skin tissue engineering)^23^, differentiation (e.g., towards an osteogenic lineage)^24^, and in early tissue organ bud (organoid) generation, condensation and maturation *in vitro* for various tissues^25^. In the latter study in particular, the substrate stiffness was as a key factor that may limit the co-culture ability to condense, mature and subsequently integrate *in vivo*. Our results further indicate the added value of microscale data over macroscale gross average mechanical measurements when characterizing such biomechanical interactions. Based on these microscale studies, we developed quantifiable parameters (RCR, MMR and IR) as indicators of tissue assembly and maturation processes. These descriptive statistics represent numerically the effects of each culture cell–ECM interaction at a population level, which is reported herein for the first time, to the best of our knowledge. Furthermore, based on all three newly suggested characterizing parameters, the values measured for co-cultured constructs resembled those of native tissue, establishing a synergistic effect of co-culture over the mono-cultures’ individual contributions towards new tissue formation.

Tissue generation during development, as well as tissue regeneration following injury (as opposed to repair mechanisms), have long been suggested to display similar characteristics that are based on the dynamic reciprocity between cells and the ECM^8,26^. Other publications demonstrated that comparable processes are also involved in cancer progression, particularly during early tumor development and subsequent maturation^27,28^. For example, correlation of microscale AFM measurements to histological analyses of early tissue formation, and subsequent maturation during cancer metastasis, displayed bi-modal distribution of Young’s moduli that are similar to our findings^28^. Understanding the contribution of each player to the biomechanics of tissue formation in this complex *in vivo* setting, however, is extremely challenging, necessitating the development of adequate model systems such as reported herein. Likewise, while current state-of-the-art in tissue engineering involves the use of various cells and biomaterial scaffolds—to restore, improve or maintain tissue function^29^—determining the optimal construct performance usually relies on tedious *in vivo* testing^30^. Thus, in addition to basic science characterization studies, the model system reported herein can offer a fine-tuning mechanism that may be implementable in engineered construct design and possibly predict their successful biomechanical modulation and integration *in vivo* in the context of regenerative medicine.

The gross mechanical tensile test characterization, performed in this study, revealed that cell removal from the native tissue during decellularization resulted in a similar, yet stiffer, biomechanical profile of isolated pcECM. Solid tissues (and also engineered tissue grafts) are usually stiffer than the cells comprising them, representing the major contribution of their ECM component to the tissue’s passive mechanical support. Therefore, every cell—whether recruited or seeded—upon first interaction with an existing or developing tissue, *e.g*., via metastasis or following cell or graft implantations, encounters an initial high mechanical mismatch with its hosting niche. Depending on the cell type and the niche, such a mismatch may be reconciled through ECM remodeling and subsequent cell integration. It is, therefore, imperative to study cell crosstalk with stiffer ECM niches at early stages of interaction. Furthermore, we demonstrated here that the adverse effect of decellularization on the mechanical properties of the resulting pcECM are reversible upon recellularization with human cells. This phenomenon is also a cell-dependent one whereby hMSCs have greater impact than HUVECs. This can be attributed to the natural role of hMSCs as reparative cells having an evolutionary conserved ECM remodeling capability to enable connective tissue formation^31^.

Other crucial biomechanical characteristics of natural ECM are those of viscoelasticity and strain stiffening, suggesting that the more cells deform the ECM, the stiffer it becomes^2,32^. Strain stiffening, in particular, may also imply that cells can initiate a dynamic process in which ECM materials of lower modulus are preferred during early tissue organization, e.g., in maintaining stem cell potency^33^, and cardiomyocyte function^34^. Subsequently, localized cell stiffness can increase due to cell–ECM remodeling, and better integration, as occurs during tissue maturation. In our study all samples tested displayed similar strain stiffening macro-mechanical characteristics, but, to fully characterize the localized cell contributions, microscale mechanical measurements were performed.

The measured Young’s modulus values of our AFM measurements were lower by two to three orders of magnitude from their equivalent tensile testing macro-mechanical characteristics, which can be attributed to inherent differences between the two testing methods. While tensile tests measure the average longitudinal forces of the entire tissue comprising cells and ECM, AFM measures the localized and perpendicular Young’s moduli at the construct surface containing either bare ECM or cells embedded within the ECM—at various stages of ECM integration and different levels of penetration. Thus, the Young’s modulus distributions (measured by AFM) suggest that at early stages of cell interactions with its niche, both components, i.e., cells and ECM, are initially separated and maintain their overall individual characteristics. Surprisingly their co-culture, though, does not show an additive or combinatory effect but rather a synergistic one, which approximated the native tissue bi-modal distributions with similar inter-peak relations. Indeed, we have previously reported on the specific synergism between hMSCs and HUVECs with regard to cell survival and proliferation on the pcECM, which obeys the Lotka–Voltera model for describing prey–predator relations in closed ecological niches^35^. The mRNA data in this study further indicated that the major contribution of hMSCs is primarily to mesenchymal tissue-like ECM organization, while endothelial cells alone are the ones required in order to enable cardiac related recognition. The co-culture of both modifies the combined gene expression so that the specific mesenchymal and cardiac tissue recognition is downregulated while the cells begin to express more genes that facilitate early tissue development and remodeling.

In conclusion, the reported novel model system enables us to better understand the dynamic reciprocity of cells and their niche during early tissue formation and maturation, thereby, expanding our toolbox for studying the key mediators and possible basic mechanisms involved. Of note is that currently, the AFM methodology employed is limited to early tissue formation processes, as attempts to study longer term cultures resulted in cell remodeling by hMSCs and shrinkage such that sample preparation could cause undesired measurement artefacts. Nevertheless, the application of this simple yet robust system for *in vitro* modeling studies, as well as the statistical and gene set analyses presented may impact basic and translational research in developmental biology, materials science, tissue engineering, regenerative medicine, and even cancer. Given the paucity of publications documenting the cell contribution to newly formed tissues’ biomechanical interaction at the cellular scale, additional research employing this model should be performed, for instance, with other cell and ECM types and more comprehensive expression profiling.

## Methods

### Cell cultures

HUVECs stably expressing the green fluorescent protein (HUVECs-GFP) were a kind gift from Prof. Gera Neufeld, as previously published^15^. These cells were sub-cultured with complete endothelial cell growth medium (EGM™, Lonza, Switzerland) on 0.2% gelatin (Sigma-Aldrich, St. Louis, MO) coated tissue culture plates. Primary bone marrow derived hMSCs stably expressing the red fluorescent protein (hMSCs-RFP, Olaf Pharmaceuticals, Worcester, MA) were sub-cultured in complete α-MEM media (Biological Industries, Israel) containing 10% fetal bovine serum (FBS, Gibco™, ThermoFisher Scientific, Waltham, MA) and 10ng/ml basic fibroblast growth factor (bFGF, added every other day; Promega, Madison, WI). All cell cultures were supplemented with an antibiotic-antimycotic solution (1%, Gibco™, ThermoFisher Scientific, Waltham, MA). Culture media were replaced every other day and appropriate splitting was performed at 70–80% confluency levels.

### Sample preparation

Acellular pcECM samples (15 × 10 × 1.5 mm, Fig. 1a) were produced as previously reported^13^. Briefly, we used a three-step protocol involving alternating a hyper-hypotonic solution (1.1% and 0.7% NaCl, respectively), a trypsin (0.05%w/v)-EDTA (0.02%w/v) solution and 1% (v/v) triton™-x-100 (in 0.1%v/v ammonium hydroxide) PBS solution. All decellularization reagents were purchased from Sigma-Aldrich (St. Louis, MO). Prior to experimentation, sliced matrices were disinfected with 70%v/v ethanol and washed with PBS containing double antibiotic-antimycotic concentration (2%v/v, Gibco™, ThermoFisher Scientific, Waltham, MA) followed by immersion in cell culture media overnight in 37 °C and 5% CO_2_^14^. For sample re-cellularization, HUVECs-GFP, hMSCs-RFP, or a co-culture thereof (1:1 ratio) were seeded in a total seeding density of 5 × 10^5^ cells/cm^2^, and were cultivated for three days in EGM™, α-MEM or a 1:1 mixture thereof, respectively. Culture media was changed every other day. To monitor cell viability and localization on the reseeded scaffold, samples were soaked in 10%v/v AlamarBlue (ThermoFisher Scientific, Waltham, MA) for 3 hours (37 °C and 5% CO_2_). Presence of cells was visible by their metabolic reduction of the AlamarBlue reagent, changing its color from dark blue to purple-pink in areas that contained viable cells in sufficient density. Acellular pcECM matrices and native left ventricular tissue slices of similar dimensions were used as negative and positive controls in all assays, respectively.

### Microscopic evaluation of sample organization and ultrastructure

For histological analyses, other three representative samples of each experimental group were fixated in fresh paraformaldehyde (4%w/v solution, PFA, Sigma-Aldrich, St. Lous, MO) for 2hrs, and transversely cut into two halves. One half of each sample was processed for paraffin blocks while the other was immersed in a sucrose 30% (w/v) solution in PBS until sample sinking. The sucrose immersed sample was subsequently embedded in Tissue-Tek optimal cutting temperature (OCT) compound (Japan). Both halves were oriented in their respective tissue blocks perpendicular to the block surface to enable cross-sectional evaluation of cell–ECM interaction. Paraffin sections (5 µm) were processed for Masson’s trichrome (MTC) and hematoxylin and eosin (H&E) stains as previously published^18^. Cryo-sections (10 µm) were fixated to positively charged slides using ice cold methanol (Sigma-Aldrich, St. Louis, MO) at 4 °C for 20 min, followed by air drying in a chemical hood in the dark for 20 min. Slides were counter-stained with NucBlue live ReadyProbes reagent (ThermoFisher Scientific, Waltham, MA) and mounted with Fluoromount-G (SouthernBiotech, Birmingham, AL). All slides were imaged using an inverted fluorescent microscope (Eclipse Ti-S, Nikon, Japan) using identical imaging parameters for all groups. Representative images are shown out of n >3 images taken per each slide.

To visualize their ultrastructure, samples were gently washed with phosphate buffered saline (PBS), fixated using 4% PFA (2hr at 4 °C) and dehydrated by ascending ethanol concentrations (70, 80, 90 and 100%), followed by short incubation in hexamethyldisilazane (HMDS, 5 min) and air drying. Scaffolds were gold sputtered for 30 s at 18 mA (JEOL JFC-1600 Auto Fine Coater, Japan) for imaging at 3 kV beam voltage and 15 mm working distance using a JSM-6360 scanning electron microscope (SEM, JEOL, Japan).

### Uniaxial testing for gross biomechanical characterization

Macroscale mechanical properties of the native heart tissue, pcECM and re-cellularized pcECM (n = 4 samples for each group) were evaluated using an Instron 5567 Universal Testing Instrument, and analyzed with BlueHill Materials Testing Software (Instron, Norwood, MA). All specimens were preconditioned with 10 cycles of tensile loading at a rate of 0.05 mm/s to 15% strain, followed by same-rate unloading. Following this, three additional cycles of tensile loading-unloading were recorded and used for analyses of the maximal stress reached at 15% strain and energy dissipation (area blocked within the ‘hysteresis loop’ curves). Each sample then went through a stress–relaxation assay in which samples were stretched at a rate of 0.5 mm/s to 20% strain and held at that constant displacement for 10 min to obtain stress–relaxation data and evaluate the maximal tensile stress, stress change (delta) during relaxation and time for relaxation of 0.5 N (Table 1). Last, tensile failure profile was evaluated by stretching the sample until failure, at a rate of 0.05 mm/s and calculating the Young’s modulus, the yielding stress and the ultimate tensile stress (UTS). All calculations were based on the automatic built-in algorithms of the BlueHill Materials Testing Software (Instron, Norwood, MA).

### Atomic force microscopy (AFM) characterization of microscale biomechanics

Tipless all-in-one cantilevers (AIOAl-TL) with a nominal spring constant of 0.2 N/m (0.07 N/m to 0.4 N/m) were purchased from BudgetSensors (Bulgaria) and modified with 4 µm (nominal diameter) polystyrene beads (Baseline Chromtech, China) according to the AFM manufacturer (JPK, Germany). Briefly, a water solution of the beads was spread on a glass cover slip, dried out and placed under microscope (Carl Zeis LSM 700, Germany). A thin line of slow-setting epoxy glue was placed on another cover slip end and the cantilevers were lowered until their tips touched the glue layer, followed by removal of excess glue on a clean glass surface. Glue-covered cantilever tips were placed on the dry beads and lifted immediately after cantilever bending was observed. Such prepared cantilevers were left to dry for 24hrs. Correct orientation and bead placement were verified individually for each tip using phase and scanning electron microscopy as well as cantilever stiffness calibration.

Samples for AFM measurement were glued on their distal sides (relative to the seeded surface) to glass microscope slides using Elmer’s superfast epoxy cement (Elmer’s Products, USA). The glass slides were then affixed to a petri dish filled with Dulbecco’s Phosphate Buffered Saline (DPBS, Life Technologies, USA) for immediate AFM scan (NanoWizard 3 AFM system, JPK, Germany). Force spectroscopy mapping was used to measure average Young’s modulus for at least n = 4 samples per group, with at least seven regions of interest (ROI) per sample, and three measurement repetitions in each ROI, using grids of 10 × 10 µm with resolution of 16 × 16 landing points per ROI. ROIs were randomly defined by an upright optic system connected inline on top of the AFM system such that every ROI contained representative morphological features (e.g., high and low density ECM, cell populated areas as indicated by AlamrBlue™ tracing within reseeded samples). As indentation set point of 50–90% of the landing force value was used throughout all experiments. Successful landing and indentation were monitored by checking force/distance curves during the experiment. Force maps were analyzed with JPKSPM Data Processing software (JPK, Germany). Young’s modulus values were calculated for every force/distance curve. Automatic Hertz model fitting was used for spherical indenter with a diameter of 4 µm and final values were calculated using the following equations (Eqs 1 and 2):

1 $$F=\frac{E}{1-{v}^{2}}[\frac{{a}^{2}+{R}_{S}^{2}}{2}ln\frac{{R}_{S}+a}{{R}_{S}-a}-a{R}_{S}],$$

2 $$\delta =\frac{a}{2}ln\frac{{R}_{S}+a}{{R}_{S}-a}$$

where ‘F’ represents force, ‘E’ – Young’s modulus, ‘ν’ – Poisson’s ratio, ‘δ’ – indentation (tip sample separation), ‘a’ – radius of contact circle, and ‘R_S_’ – radius of tip sphere.

### Phenotyping Cell–ECM interactions within the recellularized pcECM niches

Three days post seeding, reseeded pcECM samples (hMSCs, HUVECs and their co-cultures, n=6 for each sample type) were snap frozen in liquid nitrogen. Every two samples were pooled together for mRNA isolation using 700 µl QIAzol lysis reagent (Qiagen, Netherlands) and homogenized in sterile cold microtubes containing 3 mm zirconium beads (3–4 min, 3000 Hz) using the BeadBug microtube homogenizer (Benchmark Scientific, Edison, NJ). The mRNA fraction was subsequently purified using RNeasy mini columns (Qiagen) according to the manufacturer’s instructions. Isolated mRNA from equivalent cell pellets (7.5 × 10^5^ cells per pellet, n = 6 pellets for each cell type) was used as reference for basal gene expression on tissue culture plate. mRNA quality was assessed by measuring the optical density (OD) at 260/280 nm (nucleic acids/protein ratio), 260/230 nm (nucleic acids/guadinine salts), nucleic acids concentration (Nanophotometer, Implen, Germany) and the RNA integrity number (RIN value, 2100 Bioanalyzer System, Agilent Technologies, Santa Clara, CA). Only mRNA samples with a RIN >7 and 1.8< OD <2.1 were further used for reverse transcribed quantitiative realtime PCR (RT-qPCR) analyses. mRNA samples were converted to cDNA using the RT^2^ first strand kit (Qiagen) and were subsequently evaluated by qPCR (BioRad CFX96 Analyzer, BioRad, CA) using the Qiagen Human Extracellular Matrix and Adhesion Molecules PCR Array (PAHS-013ZD, Qiagen). Data pre-processing and analyses were performed using the GenEx Enterprise package ver. 6.0.3.415 (MultiD, Sweden) for interplate calibration, automatic imputation, data validation (95% validity rate), housekeeping gene and reference sample normalization, logarithmic transformation and autoscaling. The analyses included Tukey–Kramer’s post hoc corrected ANOVA with multiple t-test comparisons at an overall type 1 error of *p* < 0.05 (corrected α = 0.00061 per individual test). Significantly, up or downregulated genes that were top differentially expressed between individual sample comparisons (Supplementary Tables S1–S3) were inputted into the GeneAnalytics gene set online analyses suite (http://geneanalytics.genecards.org/) as previously published^36^. The scored results were used to generate per-group specific vector data that was subsequently plotted using principal component analyses (PCA, OriginPro 2015 16 bit Sr2 b9.2.272, OriginLab Corp., Northampton, MA).

### Statistical analyses

For all microscopy observations, representative images are presented. All quantified results are expressed as the mean ± SD or mean ± SE (for analyses of variance, ANOVA) of all biological repetitions as stated in the text. Calculated Instron and mRNA data were normally distributed (JMP statistical software, SAS, Cary, NC); thus, statistical significance in the differences of means was evaluated by one-way ANOVA with Tukey’s post hoc correction (p < 0.05 was considered significant). For AFM, Young’s modulus mapping, non-normal and bi-modal distributions were observed in all samples; thus, histogram peak fitting was performed individually for each peak (OriginPro 2015 16 bit Sr2 b9.2.272, OriginLab Corp., Northampton, MA) as detailed in the supplementary material. Based on these fittings, we developed three descriptive statistics: relative contribution ratio (RCR), mechanical mismatch ratio (MMR) and integration ratio (IR) to represent and quantify the relationship between the two peaks of the bi-modal distributions obtained (representing cell and ECM contributions, respectively) as detailed in the supplementary information.

### Data availability statement

All datasets generated and materials used and/or analysed during the current study are available from the corresponding author on reasonable request.

## Electronic supplementary material

Supplementary Information
