# Supplementary material for: Biological and mechanical interplay at the Macro- and Microscales Modulates the Cell-Niche Fate
Source: Sci Rep. 2018 Mar 2;8:3937. doi: 10.1038/s41598-018-21860-6 (PMC5834609; doi:10.1038/s41598-018-21860-6)
Supplement: Supplementary file 1 — Supplementary Information [file 41598_2018_21860_MOESM1_ESM.pdf]

Biological and mechanical interplay at the Macro- and Microscales Modulates the Cell-Niche Fate

Udi Sarig[1], Hadar Sarig[1], Aleksander Gora[2] , Muthu Kumar Krishnamoorthi[1], Gigi Chi Ting Au-Yeung[1], Elio de Berardinis[1], Su Yin Chaw[1], Priyadarshini Mhaisalkar[1], Hanumakumar Bogireddi[1] , Seeram Ramakrishna[2], Freddy Yin Chiang Boey[1], Subbu S. Venkatraman[1], Marcelle Machluf[2,3,*]

[1] School of Materials and Science Engineering (MSE), Nanyang Technological University (NTU), Singapore
[2] Department of Mechanical Engineering, National University of Singapore (NUS), Singapore
[3] Faculty of Biotechnology and Food Engineering, Technion—Israel Institute of Technology (IIT), Haifa, Israel
* Corresponding author:
Marcelle Machluf (PhD), Dean – Faculty of Biotechnology and Food Engineering | Benno Gitter and iLana Ben Ami Chair in Biotechnology | President, Israel Chapter of the Controlled Release Society |Head of the Laboratory for Cancer Drug Delivery & Cell Based Technologies. Technion—Israel Institute of Technology. Haifa, Israel Tel: +972-4-829-3068/9 website: http://drugcelltherapy.net.technion.ac.il/ | E-mail: machlufm@tx.technion.ac.il

Detailed statistical analyses for bi-modal distribution function fitting

Distribution function fittings, for each peak individually, were ranked using the Akaike information criterion (AIC), χ^2 goodness of fit and adjusted R^2 values (OriginPro 2015 16bit Sr2 b9.2.272, OriginLab Corp., Northampton, MA). Successful fit was considered as having the minimal AIC value with a $\chi^2<0.05$ and adjusted $R^2>0.95$. Among all models tested, the exponentially modified Gauss model (GaussMod) was found to provide the optimal fit for all samples and was, therefore, used for subsequent data analyses and interpretation. The GaussMod function is given by the following function (Eq. 1):

Eq. 1. $$f(x) = y_0 + \frac{A}{t_0} e^{\frac{1}{2}\left(\frac{w}{t_0}\right)^2 - \frac{x-x_c}{t_0}} \int_{-\infty}^{z} \frac{1}{\sqrt{2\pi}} e^{-\frac{y^2}{2}} dy,$$

whereas 'Y_0' is the function offset value, 'A' represents the area under the curve. 'x_c' is equivalent to the distribution center, 'w' is the distribution width and both 'x_c' and 'w' as well as t_0 are determined by the following relationships of z, distribution mean '$\bar{x}$' and its standard deviation ('SD') in Eq. 2, Eq. 3, and Eq. 4, respectively:

Eq. 2. $$z = \frac{x-x_c}{w} - \frac{w}{t_0}$$

Eq. 3. $$\bar{x} = x_c + t_0$$

Eq. 4. $$SD^2 = w^2 + t_0^2$$

Given the bi-modal distributions, descriptive statistics are provided for each peak individually, as well as additional non-conventional descriptive statistics that we developed and found to best describe the relationships between the two peaks (P_1 and P_2, which obey $\bar{x}_{P1} < \bar{x}_{P2}$). The non-conventional descriptive statistics, developed for the quantification of the inter-peak relations, are the 'Relative Contribution Ratio' (RCR, Eq. 5), 'Mechanical Mismatch Ratio' (MMR, Eq. 6) and 'Integration Ratio' (IR, Eq. 7):

Eq. 5. $$RCR = \frac{A_{P1}}{A_{P2}}$$

Eq. 6. $$MMR = \frac{\bar{x}_{P2}}{\bar{x}_{P1}}$$

Eq. 7. $$IR = \frac{\bar{x}_{P2} - \bar{x}_{P1}}{SD_{P1} + SD_{P1}}$$

These three parameters represent the relationship between the first red peak, which is attributed to the major cell contribution (as discussed in text), and the second blue peak attributed to the major pcECM mechanical contribution.

Supplementary Fig. S1. AFM set-up for microscopic mechanical evaluation of the pcECM, recellularized and native tissue. A scheme of the wet AFM set-up (**a**), where a polystyrene bead was attached to the cantilever for measurement of Young's moduli using the Hertz model (**a, inset**). Correct bead attachment was verified by SEM imaging (**b**). Force spectroscopy mapping of Young's moduli (n=4-5 samples per group) was performed on at least seven randomly selected regions of interest (ROI) per sample (black squares for size illustration purposes in **c** and **d**), and three measurement repetitions in each ROI. Care was taken in the random choice of ROI covering all representative structures and morphologies. For example, apparent differences in collagen (**c**, arrows point to high density collagen in representative pcECM sample) and cellular (**d**, arrows point to high density cells marked by reduced AlamarBlue in pink) densities. Each ROI (100µm^2) was divided by a grid of 256 spatial contact points, for each of which, the tip-sample attraction and repulsion forces as a function of tip-sample distance were recorded. The Young's modulus values determined for each measurement were averaged for each contact point resulting in a color heat map of average moduli for each ROI (**e**). Representative output from an individual point of contact force-distance curve showing approach (red line), retraction (blue line) and the fitted Hertz model (green) from which the Young's moduli value is determined (at the intersection of the horizontal and vertical dashed lines, **f**).

Supplementary Fig. S1 **Sarig et al., 2017**

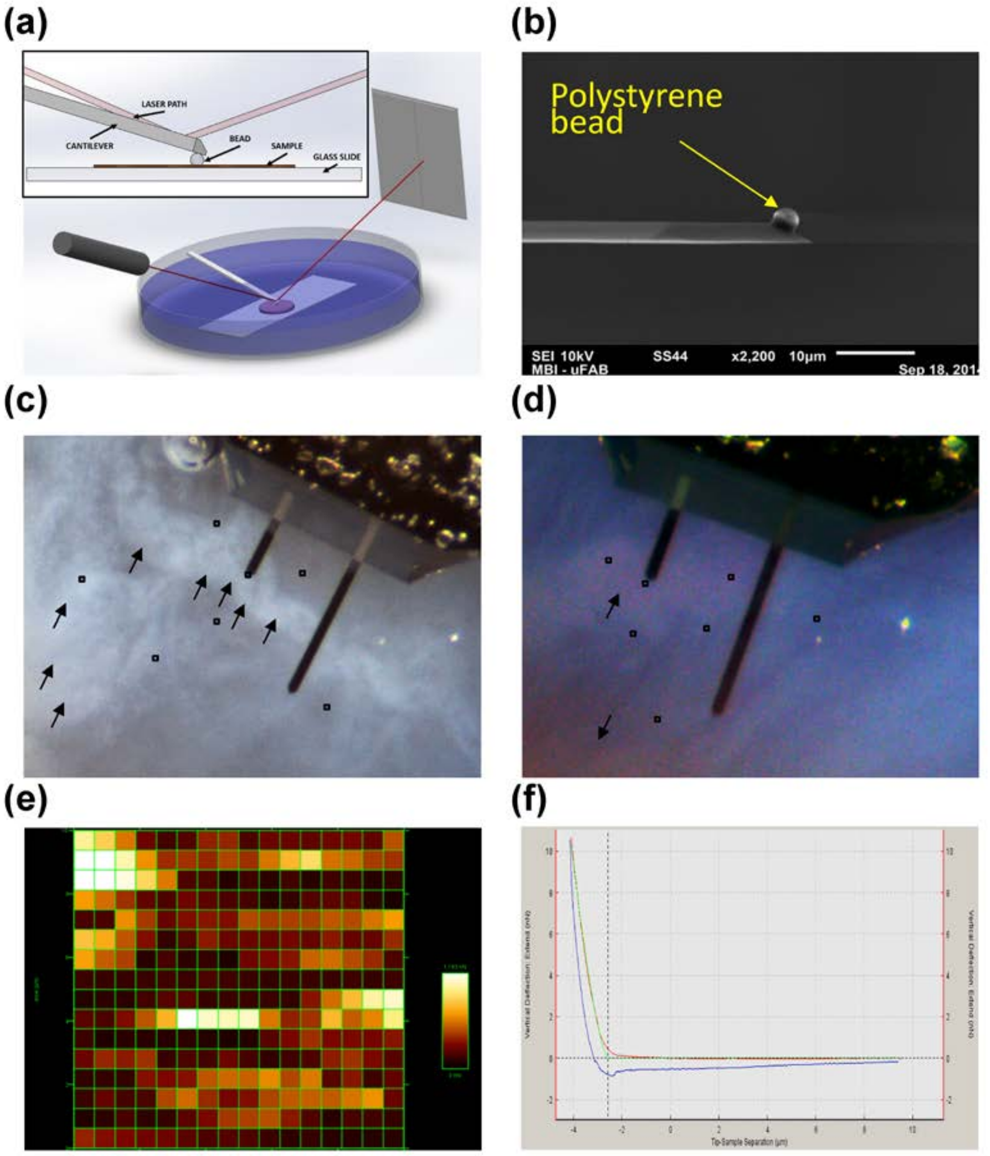

Supplementary Tables

Supplementary Table S1: Statistical analyses of differential gene expression in double normalized hMSCs vs. HUVECs comparison (correlating to Fig. 7a)

Gene name*	Fold change	Log_2(Fold Change)	*p*-value**
**CTNND2**	**38700299414913.6**	**45.13741**	**0.00000001**
**VCAN**	**8723.35065**	**13.09067**	**0.000151449**
**TGFBI**	**1881.06415**	**10.87733**	**0.000001281**
**THBS2**	**1574.48759**	**10.62067**	**0.000002051**
**COL11A1**	**1488.40926**	**10.53956**	**0.00000236**
**MMP3**	**502.85547**	**8.974**	**0.000164446**
**SPP1**	**364.97456**	**8.51165**	**0.000004573**
**COL7A1**	**346.64937**	**8.43733**	**0.00000037**
**COL1A1**	**273.23529**	**8.094**	**0.000000511**
**TNC**	**220.05218**	**7.7817**	**0.00001276**
**COL12A1**	**189.66811**	**7.56733**	**0.000009232**
**COL6A2**	**154.41474**	**7.27067**	**0.000001705**
**MMP8**	**149.15481**	**7.22067**	**0.000318693**
**TIMP3**	**144.74135**	**7.17733**	**0.00003679**
**TIMP1**	**126.58828**	**6.984**	**0.000000647**
**ITGA4**	**111.48198**	**6.80067**	**0.000101871**
**CD44**	**77.92423**	**6.284**	**0.000003091**
**ITGB2**	**44.45097**	**5.47414**	**0.000008832**
**COL16A1**	**43.1314**	**5.43067**	**0.000035588**
**ITGA1**	**36.60579**	**5.194**	**0.000034561**
**HAS1**	**31.72027**	**4.98733**	**0.000051086**
**MMP13**	**20.75321**	**4.37526**	**0.000061694**
**COL6A1**	**20.73506**	**4.374**	**0.000019477**
**MMP12**	**20.6872**	**4.37067**	**0.000088511**
**ECM1**	**12.67576**	**3.664**	**0.000126353**
**ITGB5**	**10.15417**	**3.344**	**0.000445663**
**COL5A1**	**7.13048**	**2.834**	**0.000037777**
ITGA2	4.19111	2.06733	0.000024221
LAMA2	4.14297	2.05067	0.000213475
ICAM1	3.64016	1.864	0.000263945
MMP2	3.40424	1.76733	0.00002895
SPARC	2.36854	1.244	0.000160787
ITGA3	2.06194	1.044	0.00021634
CTNND1	-2.40494	-1.266	0.000040458
**MMP15**	**-6.92909**	**-2.79267**	**0.000022807**
**ITGA6**	**-12.78163**	**-3.676**	**0.000033297**
**PECAM1**	**-566.52664**	**-9.146**	**0.000016564**
LAMA1	77.38596	6.274	0.000652824
VTN	38.96211	5.284	0.000791812
ANOS1	23.76336	4.57067	0.000836877
COL15A1	18.81746	4.234	0.006306298
ITGA7	11.26675	3.494	0.003276084
NCAM1	7.5422	2.91499	0.000849779
LAMA3	7.3649	2.88067	0.003539955

Table 1 - continued

Gene name	Fold change	Log_2(Fold Change)	*p*-value
MMP9	5.71202	2.514	0.037189645
COL14A1	3.94675	1.98067	0.008667999
ITGB1	3.20576	1.68067	0.001845391
ITGB3	3.15433	1.65733	0.002117766
MMP14	2.84942	1.51067	0.001870946
ADAMTS13	2.56804	1.36067	0.0078456
FN1	2.25636	1.174	0.027465057
TIMP2	1.56266	0.644	0.006546757
COL4A2	1.50594	0.59067	0.006968409
SGCE	1.40834	0.494	0.034494224
ITGA5	1.17338	0.23067	0.002394059
CTNNA1	-1.37809	-0.46267	0.019288085
MMP16	-1.65023	-0.72267	0.026126235
CTNNB1	-1.65787	-0.72933	0.006923804
MMP11	-1.69271	-0.75933	0.018313669
CLEC3B	-2.6378	-1.39933	0.006869969
VCAM1	-3.47256	-1.796	0.001724097
MMP1	-5.08413	-2.346	0.002241524
THBS1	-6.00432	-2.586	0.013501825
CTGF	-9.73153	-3.28267	0.009803995
ITGB4	-16.44222	-4.03933	0.001125976
SELP	-45.12953	-5.496	0.001903307
SELL	-383.38941	-8.58267	0.000847255
CDH1	44.48868	5.47537	0.160321143
SELE	14.22333	3.83019	0.781266756
ITGAM	8.28595	3.05067	0.338307823
ITGAL	4.79213	2.26067	0.243873576
CNTN1	3.67112	1.87622	0.201374738
ADAMTS8	3.52429	1.81733	0.589584753
THBS3	1.38255	0.46733	0.057771668
COL8A1	1.26342	0.33733	0.402021918
LAMB1	1.13603	0.184	0.224500069
ITGAV	1.09987	0.13733	0.618610772
LAMC1	1.07475	0.104	0.338719686
MMP10	-1.09885	-0.136	0.824188373
SPG7	-1.26225	-0.336	0.11007453
ADAMTS1	-1.3941	-0.47933	0.648121963
ITGA8	-1.84378	-0.88267	0.234622281
LAMB3	-2.71823	-1.44267	0.0813559
MMP7	-5.16702	-2.36933	0.05605597

(*) Bold and underlined data entreis represent genes that were both significantly up or down regulated (as indicated by their sign) and over 5-fold regulation in either direction, and were therefore used for the GeneAnalytics analyses. (**) Green color represents statistically significant changes, yellow—nonsignificant changes based on Tukey's post hoc correction, but still of $p<0.05$. Pink— nonsignificant changes ($p>0.05$ per individual t-test).

Supplementary Table S2: Statistical analyses of differential gene expression in double normalized coculture vs. hMSCs comparison (correlating to Fig. 7b)

Gene name*	Fold change	Log_2(Fold Change)	*p*-value**
**PECAM1**	**477.49219**	**8.89933**	**0.000024858**
**TNC**	**5.5764**	**2.47933**	**0.000463675**
ITGA5	1.88252	0.91267	0.000066889
VCAM1	-1.90176	-0.92733	0.000037808
SGCE	-1.90176	-0.92733	0.000407157
ITGB1	-1.99631	-0.99733	0.00011262
MMP10	-2.0195	-1.014	0.000429413
TGFBI	-3.08228	-1.624	0.000559934
COL5A1	-3.19836	-1.67733	0.000493377
TIMP1	-3.35737	-1.74733	0.000022312
THBS2	-4.57572	-2.194	0.000538248
ITGA2	-4.74804	-2.24733	0.000069955
**VCAN**	**-6.8717**	**-2.78067**	**0.000095878**
**ITGA4**	**-10.17766**	**-3.34733**	**0.000217823**
**VTN**	**-15.31988**	**-3.93733**	**0.000491769**
**ITGA1**	**-17.8024**	**-4.154**	**0.00001921**
**COL12A1**	**-26.24553**	**-4.714**	**0.000034715**
**COL11A1**	**-561.83395**	**-9.134**	**0.000504913**
**CTNND2**	**-1571801264845.68**	**-40.51556**	**0.000085494**
SELL	16.59488	4.05267	0.018142394
MMP7	14.58082	3.866	0.013007305
MMP15	7.81367	2.966	0.001718124
MMP13	7.40928	2.88933	0.007862843
MMP11	6.04608	2.596	0.010902245
ITGA6	5.17898	2.37267	0.002422735
NCAM1	2.76255	1.466	0.011880467
ITGA7	2.67462	1.41933	0.013962295
MMP14	2.48975	1.316	0.001171851
MMP1	1.967	0.976	0.0073958
ADAMTS1	1.93545	0.95267	0.037854623
COL6A2	1.86952	0.90267	0.019690844
CTNNB1	1.8609	0.896	0.001385541
COL6A1	1.77686	0.82933	0.04223305
THBS1	1.56844	0.64933	0.012660499
CTNND1	1.52203	0.606	0.020942513
LAMB1	1.41683	0.50267	0.040428358
FN1	1.35598	0.43933	0.011218002
CTNNA1	1.20804	0.27267	0.037872594
ITGAV	-1.499	-0.584	0.0055922
MMP16	-1.55905	-0.64067	0.006392067
COL4A2	-1.87125	-0.904	0.003581326
ECM1	-2.22016	-1.15067	0.005284869
COL7A1	-2.42391	-1.27733	0.004195856
COL16A1	-3.24301	-1.69733	0.000881218

Table 2 - continued

Gene name	Fold change	Log_2(Fold Change)	*p*-value
SPARC	-5.20777	-2.38067	0.000908794
ICAM1	-5.39143	-2.43067	0.001923286
COL8A1	-6.57656	-2.71733	0.003634194
TIMP3	-7.85712	-2.974	0.001642081
MMP12	-9.83097	-3.29733	0.027366059
ANOS1	-14.83226	-3.89067	0.000814431
ITGAL	-72.37068	-6.17733	0.007055734
ADAMTS8	25.92011	4.696	0.290324226
MMP9	5.82665	2.54267	0.220841728
ITGA8	4.51899	2.176	0.069069143
SPP1	1.88688	0.916	0.208755193
LAMA3	1.87818	0.90933	0.093183977
COL14A1	1.8184	0.86267	0.193348989
HAS1	1.81001	0.856	0.161273807
ITGB2	1.73628	0.796	0.065568816
CNTN1	1.33537	0.41724	0.74611282
LAMC1	1.3189	0.39933	0.077448261
ITGA3	1.2249	0.29267	0.178828501
COL15A1	1.22208	0.28933	0.494216052
LAMA1	1.19141	0.25267	0.325136324
SPG7	1.14817	0.19933	0.236638687
LAMB3	1.14552	0.196	0.220579793
MMP2	1.12714	0.17267	0.316459708
ADAMTS13	1.1014	0.13933	0.65407496
ITGB4	1.07128	0.09933	0.851345374
TIMP2	1.04681	0.066	0.618822849
CLEC3B	-1.02859	-0.04067	0.927906177
CTGF	-1.04295	-0.06067	0.908036156
CD44	-1.09227	-0.12733	0.694468932
ITGB3	-1.10496	-0.144	0.243536447
THBS3	-1.23456	-0.304	0.296824826
ITGB5	-1.29894	-0.37733	0.052672956
LAMA2	-1.50943	-0.594	0.06441316
MMP3	-1.67095	-0.74067	0.494898502
SELP	-1.82626	-0.86889	0.285453831
COL1A1	-3.08228	-1.624	0.081759144
ITGAM	-11.086	-3.47067	0.229023125
CDH1	-15.04765	-3.91147	0.322237586
MMP8	-18.21851	-4.18733	0.054847078
SELE	-102.31362	-6.67685	0.631840876

(*) Bold and underlined data entreis represent genes that were both significantly up or down regulated (as indicated by their sign) and over 5-fold regulation in either direction, and were therefore used for the GeneAnalytics analyses. (**) Green color represents statistically significant changes, yellow—nonsignificant changes based on Tukey's post hoc correction, but still of $p<0.05$. Pink— nonsignificant changes ($p>0.05$ per individual t-test).

Supplementary Table S3: Statistical analyses of differential gene expression in double normalized coculture vs. HUVECs comparison (correlating to Fig. 7c)

Gene name*	Fold change	Log_2(Fold Change)	*p*-value**
**VCAN**	**1269.46068**	**10.31**	**0.000395697**
**TNC**	**1227.09837**	**10.26104**	**0.000008465**
**SPP1**	**688.66193**	**9.42765**	**0.000087147**
**TGFBI**	**610.28247**	**9.25333**	**0.000002521**
**THBS2**	**344.09585**	**8.42667**	**0.000006996**
**COL6A2**	**288.6812**	**8.17333**	**0.0000053**
**MMP13**	**153.76638**	**7.2646**	**0.000244949**
**COL7A1**	**143.01275**	**7.16**	**0.000003834**
**LAMA1**	**92.1982**	**6.52667**	**0.000581362**
**ITGB2**	**77.17936**	**6.27014**	**0.00006721**
**CD44**	**71.34135**	**6.15667**	**0.000047536**
**HAS1**	**57.41411**	**5.84333**	**0.000425501**
**TIMP1**	**37.70455**	**5.23667**	**0.000001191**
**COL6A1**	**36.84338**	**5.20333**	**0.000031022**
**NCAM1**	**20.8357**	**4.38099**	**0.000024252**
**COL16A1**	**13.29981**	**3.73333**	**0.0000978**
**ITGB5**	**7.81728**	**2.96667**	**0.000513978**
**COL12A1**	**7.22668**	**2.85333**	**0.00033063**
**MMP14**	**7.09433**	**2.82667**	**0.000104127**
MMP2	3.83706	1.94	0.000141013
ADAMTS13	2.82843	1.5	0.000562562
ITGA5	2.20891	1.14333	0.000020879
**VCAM1**	**-6.60397**	**-2.72333**	**0.000328755**
**SELP**	**-82.4183**	**-6.36489**	**0.00028728**
MMP3	300.94026	8.23333	0.002213204
COL1A1	88.64701	6.47	0.000802218
ITGA7	30.13427	4.91333	0.001494881
COL15A1	22.99636	4.52333	0.006760226
TIMP3	18.42169	4.20333	0.001196766
LAMA3	13.8326	3.79	0.003563509
ITGA4	10.9536	3.45333	0.002511754
COL14A1	7.17676	2.84333	0.009633272
ECM1	5.70938	2.51333	0.001372235
CNTN1	4.90232	2.29346	0.001036817
MMP11	3.57184	1.83667	0.039235388
FN1	3.05958	1.61333	0.009284282
ITGB3	2.85469	1.51333	0.003172029
LAMA2	2.74473	1.45667	0.004346804
ITGA3	2.52567	1.33667	0.002457524
COL5A1	2.22942	1.15667	0.002098263
ITGA1	2.05623	1.04	0.028512662
TIMP2	1.6358	0.71	0.000739673
LAMB1	1.60956	0.68667	0.010185237
ITGB1	1.60585	0.68333	0.045404172

Table 3 - continued

Gene name	Fold change	Log_2(Fold Change)	*p*-value
LAMC1	1.41748	0.50333	0.038676458
COL4A2	-1.24258	-0.31333	0.042898396
CTNND1	-1.58008	-0.66	0.013049036
SPARC	-2.19872	-1.13667	0.015887984
ITGA6	-2.46798	-1.30333	0.022627362
MMP16	-2.57279	-1.36333	0.003144992
MMP1	-2.58471	-1.37	0.017779345
CLEC3B	-2.71321	-1.44	0.044475744
THBS1	-3.8282	-1.93667	0.032569121
COL8A1	-5.20537	-2.38	0.009459329
SELE	-7.19336	-2.84667	0.004893118
CTGF	-10.14948	-3.34333	0.016327219
ITGB4	-15.34823	-3.94	0.003226093
SELL	-23.10287	-4.53	0.004678186
ADAMTS8	91.35003	6.51333	0.082350392
MMP9	33.28192	5.05667	0.054340947
CTNND2	24.62162	4.62185	0.14025827
MMP8	8.18699	3.03333	0.116029701
CDH1	2.95652	1.5639	0.348014713
MMP7	2.8219	1.49667	0.110427942
COL11A1	2.6492	1.40556	0.20303527
VTN	2.54324	1.34667	0.123107512
ITGA8	2.45094	1.29333	0.226653411
MMP12	2.10429	1.07333	0.338037028
ANOS1	1.60214	0.68	0.341195565
ADAMTS1	1.38831	0.47333	0.661855504
MMP15	1.12766	0.17333	0.678305378
CTNNB1	1.12246	0.16667	0.354649307
THBS3	1.11987	0.16333	0.612377847
SPG7	-1.09936	-0.13667	0.258815078
ITGA2	-1.13288	-0.18	0.272496613
CTNNA1	-1.14076	-0.19	0.154977688
PECAM1	-1.18646	-0.24667	0.432564358
ITGAM	-1.33793	-0.42	0.915618072
SGCE	-1.35035	-0.43333	0.071895509
ITGAV	-1.36289	-0.44667	0.173621809
ICAM1	-1.4811	-0.56667	0.196829407
MMP10	-2.21914	-1.15	0.116255476
LAMB3	-2.37293	-1.24667	0.119800291
ITGAL	-15.10199	-3.91667	0.122694148

(*) Bold and underlined data entreis represent genes that were both significantly up or down regulated (as indicated by their sign) and over 5-fold regulation in either direction, and were therefore used for the GeneAnalytics analyses. (**) Green color represents statistically significant changes, yellow—nonsignificant changes based on Tukey's post hoc correction, but still of $p<0.05$. Pink— nonsignificant changes ($p>0.05$ per individual t-test).
